# High dose of psilocybin induces acute behavioral changes without inducing conditioned place preference in Sprague-Dawley rats

**DOI:** 10.1177/02698811251368361

**Published:** 2025-09-22

**Authors:** Vitor Bruno, Martha López-Canul, Brandon Richardson, Rosana Camarini, Tania Marcourakis, Gabriella Gobbi

**Affiliations:** 1 Department of Clinical and Toxicological Analysis, School of Pharmaceutical Sciences, University of São Paulo, Brazil; 2 Department of Psychiatry, McGill University and Research Institute of the McGill University Health Center, Montreal, QC, Canada; 3 Department of Pharmacology, Institute of Biomedical Sciences, University of São Paulo, Brazil

**Keywords:** psychedelics, rewarding, addiction, behavior

## Abstract

**Background:**

In recent years, there has been a resurgence of scientific interest in psychedelics, including psilocybin, for their potential in treating neuropsychiatric disorders. However, the reward-related effects of psilocybin and its impact on behavior remain underexplored.

**Aims:**

We aimed to evaluate the potential rewarding effects of high doses of psilocybin and its effects on rat behavior.

**Methods:**

Sprague-Dawley rats were exposed to the conditioned place preference (CPP) paradigm. Over an 8-day period, rats were administered either psilocybin (10 mg/kg, i.p.) or vehicle (0.9% saline, i.p.) on odd conditioning days, while receiving vehicle (0.9% saline, i.p.) on even conditioning days. The potential rewarding effect induced by psilocybin was assessed 48 hours after the last psilocybin injection. Behavioral assessments, including head twitch, body shaking, grooming, body licking, defecation pellets, and rearing, were conducted during the CPP exposure.

**Results:**

Psilocybin did not induce CPP in rats, highlighting its lack of reinforcing effects under these conditions. However, this regimen of administration led to modifications in the behavioral profile during CPP test by increasing head twitching, wet-wet-dog shaking, and defecation pellets and decreasing grooming, body licking, and rearing compared to the vehicle group. Importantly, 48 hours after the final psilocybin injection, no behavioral differences were observed between psilocybin and vehicle groups.

**Conclusion:**

Psilocybin at this regimen (10 mg/kg, every other day) does not induce CPP, but induces changes in behavior, which disappear 48 hours after the last injection. More research is needed to better evaluate the addiction liability of psychedelics using different paradigms, doses, and protocols.

## Introduction

Psychedelics are psychoactive substances known for their ability to influence perception, mood, and cognitive processes (Nichols, 2016), now undergoing renewed scientific interest and exploration in the scientific community (Murnane, 2018; Nutt, 2019), particularly within their potential therapeutic applications for mental health disorders (McClure-Begley and Roth, 2022).

Psilocybin (4-phosphoryloxy-N,N-dimethyltryptamine) has emerged as a leading candidate for therapeutic exploration due to its unique pharmacological properties. Psilocybin is a naturally occurring compound found in certain species of mushrooms, which are popularly referred to as “magic mushrooms” (Lowe et al., 2021). As a tryptamine alkaloid, it primarily targets the serotonin receptors in the brain, particularly the serotonin 2A receptors (5-HT_2A_R; Madsen et al., 2019). Upon ingestion, psilocybin is rapidly hydrolyzed into psilocin, the active compound responsible for its psychoactive effects (Dinis-Oliveira, 2017).

Research has highlighted psilocybin’s potential therapeutic benefits for conditions such as depression, anxiety, post-traumatic stress, and addiction, both in clinical and preclinical models (Bruno et al., 2022; Floris et al., 2024; Goodwin et al., 2022; Jeanblanc et al., 2024). Additionally, studies suggest that psilocybin can induce profound psychological experiences, potentially leading to lasting improvements in mood and perspective (Griffiths et al., 2011; McCulloch et al., 2022).

Unlike most other psychoactive drugs, psychedelics like psilocybin seems to do not induce rewarding (Canal and Murnane, 2017; Nichols, 2016), even if more research including studies of conditioned place preference (CPP), intravenous self-administration, and drug discrimination are needed to completely rule out this risk. Indeed, it is important to note that some research has raised concerns about the potential of some psychedelics, such as ayahuasca and 3,4-methylenedioxymethamphetamine (MDMA), as well as the dissociative anesthetic drug ketamine, to elicit rewarding effects; (Cata-Preta et al., 2018; Contó et al., 2022; Daza-Losada et al., 2007), which warrants further exploration under specific conditions and paradigms.

Fantegrossi et al. (2004) have explored the effects of psychedelics, including psilocybin, in monkeys rhesus using the intravenous self-administration, finding only a transient reinforcing property of these substances, even if the doses used in these experiments were lower than the doses currently used in clinical trials. Moreover, the CPP paradigm is a preclinical model commonly used to assess the rewarding effects of stimuli in the context of acquisition, expression, and reinstatement, by associating a specific environment with drug administration. Reward is indicated by increased time spent in the drug-paired compartment (Tzschentke, 1998). Additionally, behavioral assessments during the CPP are essential for understanding the cognitive and neurophysiological effects of drugs, as well as for further investigation of their mechanisms of action (Gevins et al., 2002).

This study aimed to examine whether four administrations of psilocybin at high doses on alternate days exhibit rewarding or aversive effects using the CPP paradigm, while also evaluating behavioral patterns. The absence of CPP or aversion, along with the consistent behavioral clustering over time, suggests that psilocybin does not produce rewarding or aversive effects. These findings strengthen the evidence that psilocybin has a low liability for abuse and support its safe and predictable use in therapeutic contexts.

## Materials and methods

### Animals

Twenty adult male Sprague-Dawley rats (225–340 g; Charles River Laboratories) were housed at 22°C with access to food and water ad libitum and maintained under a 12 hours light/dark cycle (lights on, 7:00 A.M.; lights off, 7:00 P.M.). Rats were housed in pairs which received the same treatment. All procedures were approved by the McGill University Ethics Committee and are in line with the Canadian Institute of Health Research for Animal Care and Scientific Use, the Animal Care Committee of McGill University (protocol number 5253). All efforts were made to minimize animal suffering, and the 3Rs rule (reduce, refine, replace) were applied when possible.

### Psilocybin

Psilocybin was obtained from Psygen Labs Inc. (Calgary, AB, Canada), dissolved in a 0.9% NaCl (saline) solution, and administered intraperitoneally (i.p.) at a dose of 10 mg/kg with an injection volume of 500 μL. Control group received vehicle (0.9% saline solution) at the same volume. The dose of 10 mg/kg was based on pharmacokinetics studies by Higgins et al. (2021), showing that this dose corresponds to a *C*_max_ of approximately 1106 ng/mL of psilocin. Moreover, considering Food and Drug Administration table of conversion (Nair and Jacob, 2016) and considering that rodents have less affinity than human for the 5-HT_2A_ receptors (Tan et al., 1999), the 10 mg/kg corresponds approximately to the dose used in human studies (Carhart-Harris et al., 2021; Goodwin et al., 2022).

### Conditioned place preference

**Figure 1.**
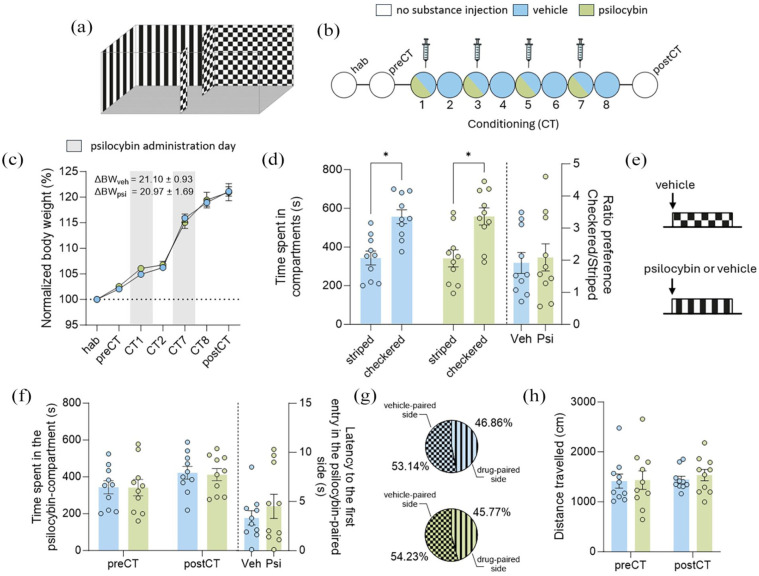
Psilocybin does not induce conditioned place preference. (a) CPP apparatus. (b) Experimental timeline (*n* = 10 each group). (c) Normalized body weight. (d, left) Time spent in the compartments on the preCT day. Two-way ANOVA with repeated measures followed Bonferroni’s test. (d, right) Ratio preference (time spent on the checkered-side compartment divided by the time spent in the striped-compartment). Unpaired Student’s *t*-test. (e) Conditioning biased paradigm. (f, left) Time spent in the psilocybin-compartment. Two-way ANOVA with repeated measure. (f, right) Latency to the first entry in the psilocybin-paired side. Unpaired Student’s *t*-test. (g) Time spent in compartments (in percentage) for each group in the postCT day. (h) Distance travelled. Two-way ANOVA with repeated measures. Data are expressed as mean ± SEM. *p < 0.05.

The CPP test was conducted in a rectangular box (63 cm × 32 cm × 35 cm) divided into two equal-sized chambers (30 cm × 30 cm × 30 cm): one with walls featuring black and white vertical stripes and the other with walls in a black and white checkered pattern. Both chambers had a grid floor (Ugo Basile, Italy), as illustrated in Figure 1(a). After each CPP round, the apparatus was wiped with a 70% ethanol solution to eliminate odor traces before testing the next animal.

All experimental days were videotaped (Sony HDR-CX405); the time spent in each compartment and the traveled distance were analyzed through the software ANY-maze Video Tracking System (Version 7.4, Stoelting Co.). The CPP score was determined by recording the total time spent in each compartment during the test session (Yates, 2023). The preference ratio was then calculated as the proportion of time spent in the preferred compartment compared to the time spent in the non-preferred compartment. Behavioral assessments were conducted while animals were in the CPP apparatus. Hallucinogenic-like behaviors (head twitches and wet-dog shakes), compulsive or obsessive-like behaviors (grooming and body licking), and rearing and defecation pellets were manually quantified by a single blinded observer. These behavioral events were analyzed considering bouts of each behavior. Table 1 provides the operational definitions used to identify and quantify each behavioral parameter.

**Table 1.** Descriptions of behavioral parameters evaluated during the CPP test.

Behavior	Description	References
Head twitch	Rapid, involuntary side-to-side movement of the head	de la Fuente Revenga et al. (2020), Halberstadt (2020), Jaster and González-Maeso (2023)
Wet-dog shaking	Vigorous, rhythmic body shake resembling a wet dog drying off	Bedard and Pycock (1977), Buchborn et al. (2023)
Grooming	Stereotyped sequence of self-directed cleaning behaviors involving face, head, body, and limbs	Brownstien et al. (2024), Gattuso et al. (2024), Smolinsky et al. (2009)
Body licking	Specific self-directed behavior involving licking of the flanks, back, and hind limbs	Smolinsky et al. (2009)
Rearing	Standing on hind legs, typically to explore the vertical space of the environment	Chen et al. (2023)

#### Protocol

Twenty rats were randomly divided into two groups. On the first day, they were habituated in the CPP apparatus to minimize novelty or exploratory effect. On the next day, a preconditioning test (preCT) was conducted to assess baseline compartment preference. During these 2 days, the CPP door remained open, allowing free access to both compartments for 15 minutes without drug administration.

A biased conditioning paradigm was applied due to their natural preference for one of the compartments. Therefore, on the third day, 10 rats received psilocybin injections on odd conditioning days (CT1, 3, 5, and 7) and were placed immediately into the non-preferred compartment, while on even conditioning days (CT2, 4, 6, and 8), they received a vehicle injection and were placed immediately into the preferred compartment. The remaining 10 rats (vehicle group) received vehicle injections on all conditioning days and were exposed to both compartments in an alternating manner, matching the psilocybin group in terms of handling, injection procedures, and environmental exposure. Additionally, the control group allowed for the interpretation of behavioral changes that may have been specifically induced by psilocybin throughout the conditioning period. Each conditioning session lasted 20 minutes, with the compartment transition door closed to confine the rats to the specific compartment.

On the 9th day, the rats were placed in the CPP apparatus with the door open to allow free exploration of the compartments for 15 minutes to assess the potential expression of psilocybin-induced CPP. After the test, rats were anesthetized with isoflurane and euthanized by decapitation. The timeline protocol is illustrated in Figure 1(b).

### Statistical analysis

A two-way Analysis of Variance (ANOVA) with repeated measures (RMs) was applied to compare body weight throughout the protocol. The body weight was normalized for each rat, considering the habituation day as the baseline. For the assessment of the basal preference for each compartment and the psilocybin-induced CPP expression test, a two-way RM ANOVA with Bonferroni’s corrected post hoc test was applied. The distance travelled on the test day was evaluated by paired Student’s *t*-test. The latency to the first entry in the psilocybin-paired compartment was assessed by unpaired Student’s *t*-test.

The behavioral assessment during the conditioning sessions was analyzed by two-way RM ANOVA with Bonferroni’s corrected post hoc test. To provide an integrated view of the overall effect of the tested drug and to explore the relationships between variables (behavioral assessment) and groups (treatment), principal component analysis (PCA) was performed. PCA is a novel statistical method that allows the analysis of multiple behavioral data points simultaneously, providing an integrated view of the overall effect of the tested drug (Greenacre et al., 2022). The principal components (PCs) were derived from eigenvalue decomposition of the correlation matrix. Components with eigenvalues greater than 1 were retained based on the Kaiser criterion. A PCA biplot was used to simultaneously visualize the scores (individuals) and loadings (behavioral assessment). To illustrate the dispersion and overlap between groups and behaviors, 95% confidence interval ellipses were overlaid on the biplot. A correlation matrix was conducted to evaluate the relationships among behavioral parameters under acute and repeated psilocybin treatment conditions. PCA and correlation matrix were performed using RStudio (version 2024.04.2+764; Posit Software, PBC, Boston, MA, USA), employing the Comprehensive R Archive Network (CRAN) packages FactoMineR and factoextra for PCA and the Hmisc and corrplot for the correlation matrix. A heat map visualizing individual performance across behaviors was generated using data normalized by their respective maximum values.

A time chart was generated in RStudio using the CRAN packages ggplot2, hms, and dplyr to visualize the temporal distribution of hallucinogen- and compulsive or obsessive-like behaviors across individuals from different experimental groups. Behavioral events were plotted as horizontal segments over time, enabling the identification of patterns in the occurrence of each behavior.

The behavioral assessment on the postCT day was analyzed by unpaired Student’s *t*-test. Statistical analyses were performed using GraphPad Prism (version 10.2.3; GraphPad Software, LLC, San Diego, CA, USA), considering a significance level of α < 0.05. Numerical data are presented as mean ± SEM.

## Results

### Psilocybin fails to induce rewarding or aversive effects in the CPP paradigm

A two-way RM ANOVA on body weight (Figure 1(c)) revealed no significant days × treatment interaction (*F*_(6, 108)_ = 0.5701; *p* = 0.05), nor a significant main effect of treatment (*F*_(1, 18)_ = 0.0740; *p* > 0.05). A significant main effect of days (*F*_(6, 108)_ = 392.9; *p* < 0.0001) indicated that psilocybin treatment did not influence the body weight of the animals.

Prior to CPP exposure, we evaluated the baseline preference of animals for each compartment. A two-way repeated-measures ANOVA revealed no significant effects for group × compartment interaction (*F*_(1, 18)_ = 0.0009; *p* > 0.05) or main effect of group (*F*_(1, 18)_ = 1.000; *p* > 0.05). However, a significant effect of compartment was observed (*F*_(1, 18)_ = 14.28; *p* = 0.0014), indicating a preference for the checkered compartment (Figure 1(d), left). The preference ratio analysis showed no significant difference in the degree of preference for the checkered compartment between the vehicle and psilocybin groups (*t*_(18)_ = 0.3221; *p* > 0.05; Figure 1(d), right). Given the baseline preference of both groups for the checkered compartment, the conditioning protocol was adjusted accordingly. On odd conditioning days, rats were confined to the striped compartment (the non-preferred chamber) following psilocybin injection. Conversely, on even conditioning days, they were confined to the checkered compartment (the preferred chamber) after vehicle injection, as illustrated in Figure 1(e).

To assess the expression of psilocybin-induced CPP (Figure 1(f), left), a two-way RM ANOVA revealed no significant effects for days × treatment interaction (*F*_(1, 18)_ = 0.0117; *p* > 0.05), main effect of days (*F*_(1, 18)_ = 3.947; *p* > 0.05) or main effect of treatment (*F*_(1, 18)_ = 0.02453; *p* > 0.05). Moreover, an unpaired Student’s *t*-test showed no difference in the latency to the first entry into the psilocybin-paired compartment (*t*_(18)_ = 0.8212; *p* > 0.05; Figure 1(f), right). The percentage of time spent in the vehicle- or psilocybin-paired compartment for each group is represented in Figure 1(g). Concerning the distance travelled (Figure 1(h)), a two-way RM ANOVA showed no significant effects for treatment × compartment interaction (*F*_(1, 18)_ = 0.0576; *p* > 0.05), treatment (*F*_(1, 18)_ = 0.2237; *p* > 0.05), or compartment (*F*_(1, 18)_ = 0.1857; *p* > 0.05). These results indicate that psilocybin did not induce either CPP or aversion.

### Behavioral changes resulting from psilocybin treatment

**Figure 2.**
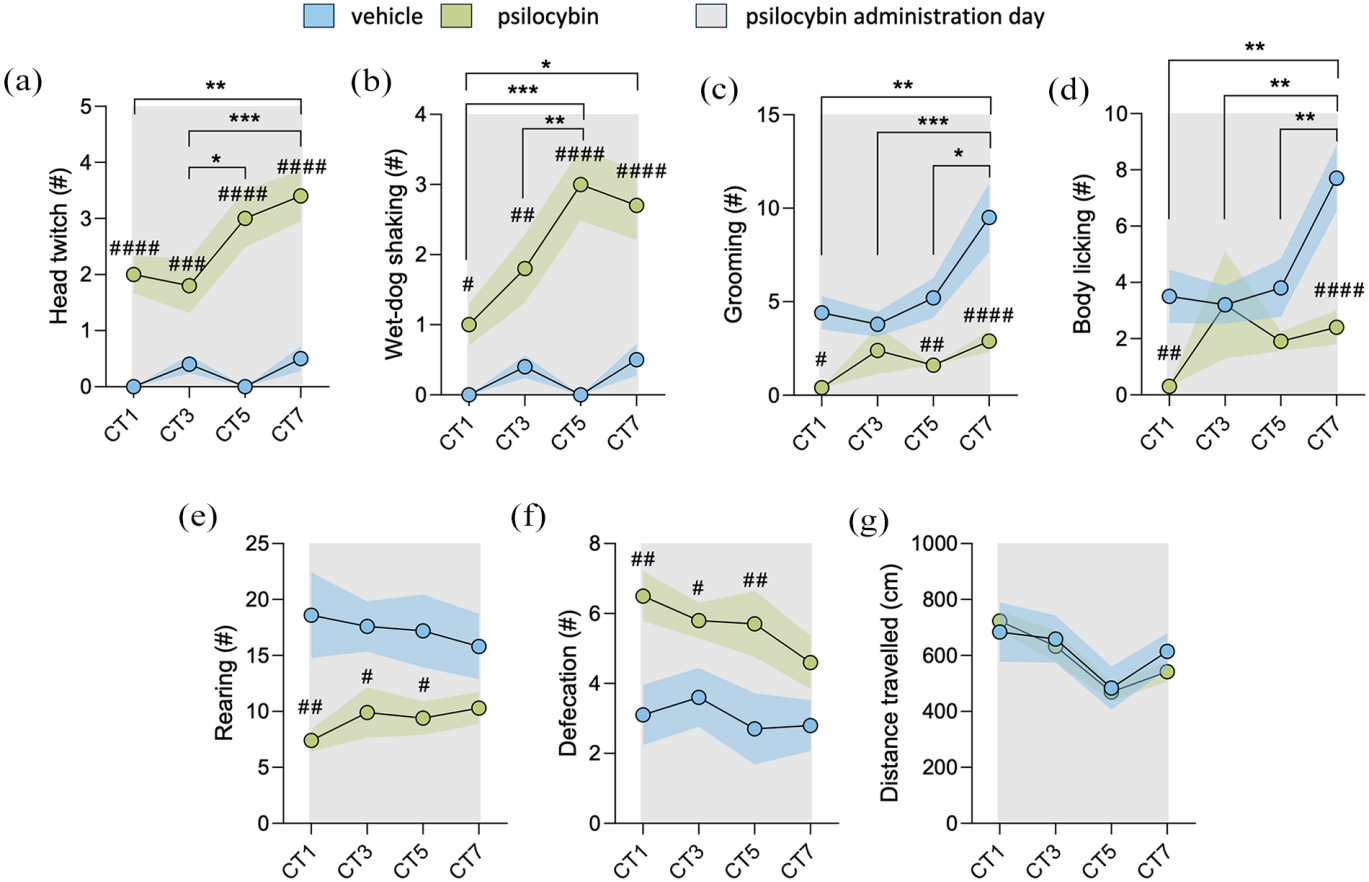
Behavioral assessment during conditioning period (*n* = 10 each group). Analysis of **(a)** head twitch, **(b)** wet-dog shaking, **(c)** grooming, **(d)** body licking, **(e)** rearing, **(f)** defecation pellets, and **(g)** distance travelled performed by the groups. Two-way RM ANOVA followed Bonferroni’s test. Data are expressed as mean ± SEM. ^*^Highlights the differences within a group, whereas # compares between groups. *p < 0.05, **p < 0.01, ***p < 0.001, ****p < 0.0001. ^#^*p* < 0.05, ^##^*p* < 0.01, ^###^*p* < 0.001, ^####^*p* < 0.0001.

Behavioral assessment was evaluated during the conditioning sessions using a two-way RM ANOVA analysis. Head twitch response analyses (Figure 2(a)) showed significant effects for days × treatment interaction (*F*_(3, 27)_ = 4.5323; *p* < 0.05) and main effect of days (*F*_(3, 27)_ = 5.989; *p* < 0.01) and treatment (*F*_(1, 9)_ = 59.83; *p* < 0.0001). Bonferroni’s test showed a higher score on head twitch performance by the psilocybin group compared to the vehicle group (*p* < 0.0001 for CT1, 5, and 7; *p* < 0.001 for CT3 days). Additionally, we observed a progressive increase in head twitch events on the psilocybin group from CT1 to CT7 (*p* < 0.01) and from CT3 to CT5 and CT7 (*p* < 0.05 and *p* < 0.001, respectively). Wet-dog-shaking analyses (Figure 2(b)) revealed significant effects for days × treatment interaction (*F*_(3, 27)_ = 4.578; *p* = 0.0102), main effects of days (*F*_(3, 27)_ = 5.713; *p* = 0.0037), and treatment (*F*_(1, 9)_ = 47.09; *p* < 0.0001). Bonferroni’s test indicated no significant differences in the amount of wet-dog shaking performed by the vehicle group throughout the conditioning period. Significant differences were found within the psilocybin group throughout the protocol, as follows: the psilocybin group performed more wet-dog shaking on CT5 and CT7 compared to CT1 (*p* < 0.001 and *p* < 0.01, respectively) and on CT5 compared to CT3 (*p* < 0.05). Additionally, the psilocybin group exhibited more wet-dog shaking than the vehicle group on CT1 (*p* < 0.05), CT3 (*p* < 0.01), CT5, and CT7 (*p* < 0.0001 for both days).

Grooming behavior analyses (Figure 2(c)) revealed no significant effects for the days × treatment interaction (*F*_(3, 27)_ = 2.957; *p* = 0.0502). However, it was found a main effect of days (*F*_(3, 27)_ = 4.664; *p* = 0.0094) and treatment (*F*_(1, 9)_ = 26.57; *p* = 0.0006). Bonferroni’s post hoc test showed an increase in grooming on CT7 compared to the previous days (CT5, *p* < 0.05; CT3, *p* < 0.001; and CT1, *p* < 0.01) within the group treated with vehicle; meanwhile, animals treated with psilocybin did not show differences in grooming throughout the days. Moreover, the vehicle group performed more grooming than the psilocybin group on CT1 (*p* < 0.01), CT5 (*p* < 0.01), and CT7 (*p* < 0.0001). Body licking behavior analyses (Figure 2(d)) showed significant effects for days × treatment interaction (*F*_(3, 27)_ = 4.015; *p* = 0.0174), days (*F*_(3, 27)_ = 4.189; *p* = 0.0148) and treatment (*F*_(1, 9)_ = 6.602; *p* = 0.0302). Bonferroni’s post hoc test revealed that the number of body licking performed by the vehicle group was higher on CT7 compared to CT5, CT3, and CT1 (*p* < 0.01 for all). On the other hand, there were no differences in the amount of body licking performed by the psilocybin group throughout the conditioning period. Consequently, the vehicle group performed more body licking than the psilocybin group on CT1 (*p* < 0.01) and CT7 (*p* < 0.0001).

Rearing behavior (Figure 2(e)) showed no differences for days × treatment interaction (*F*_(3, 27)_ = 0.5645; *p* > 0.05) and days (*F*_(3, 27)_ = 0.0601; *p* > 0.05), but a difference for treatment (*F*_(1, 9)_ = 9.682; *p* = 0.0125), indicating that psilocybin induce a reduction in rearing behavior. The number of defecation pellets (Figure 2(f)) showed no significant effects for days × treatment interaction (*F*_(3, 27)_ = 0.5403; *p* > 0.05) and days (*F*_(3, 27)_ = 1.017; *p* > 0.05), but found a main effect of treatment (*F*_(1, 9)_ = 10.44; *p* = 0.0103). Distance traveled within the compartments (Figure 2(g)) showed no effects for days × treatment interaction (*F*_(3, 27)_ = 0.2870; *p* > 0.05) and treatment effect (*F*_(1, 9)_ = 0.0647; *p* > 0.05), but a day effect (*F*_(3, 27)_ = 10.07; *p* = 0.0001). Means and SEM are displayed in Table 2.

**Table 2.** Behavioral assessment performed during conditioning days. Data are expressed as mean ± SEM.

Days	Groups	Head twitch	Wet-dog shaking	Grooming	Body licking	Rearing	Defecation	Distance travelled
CT1	Vehicle	0.00 ± 0.00	0.00 ± 0.00	4.40 ± 0.90	3.50 ± 0.96	18.60 ± 3.82	3.10 ± 0.86	683.87 ± 106.34
	Psilocybin	2.00 ± 0.33	1.00 ± 0.30	0.40 ± 0.22	0.30 ± 0.15	7.40 ± 1.01	6.50 ± 0.72	722.24 ± 35.38
CT3	Vehicle	0.40 ± 0.16	0.40 ± 0.16	3.80 ± 0.66	3.20 ± 0.68	17.60 ± 2.25	3.60 ± 0.85	659.11 ± 84.81
	Psilocybin	1.80 ± 0.49	1.80 ± 0.49	2.40 ± 1.26	3.20 ± 1.91	9.90 ± 2.26	5.80 ± 0.51	633.03 ± 54.14
CT5	Vehicle	0.00 ± 0.00	0.00 ± 0.00	5.20 ± 1.07	3.80 ± 1.03	17.20 ± 3.26	2.70 ± 1.02	483.40 ± 77.33
	Psilocybin	3.00 ± 0.52	3.00 ± 0.52	1.60 ± 0.34	1.90 ± 0.35	9.40 ± 1.51	5.70 ± 0.94	469.80 ± 31.68
CT7	Vehicle	0.44 ± 0.24	0.50 ± 0.22	9.50 ± 1.83	7.70 ± 1.17	15.80 ± 2.91	2.80 ± 0.73	614.30 ± 64.52
	Psilocybin	3.40 ± 0.45	2.70 ± 0.50	2.90 ± 0.59	2.40 ± 0.60	10.30 ± 1.43	4.60 ± 0.76	542.24 ± 35.26

Our findings indicate that psilocybin treatment progressively increased the number of head twitches and wet-dog-shaking behaviors, while simultaneously reducing the frequency of grooming, body licking, and rearing events.

### Alternate psilocybin administration induces stable and distinct behavioral clusters

**Figure 3.**
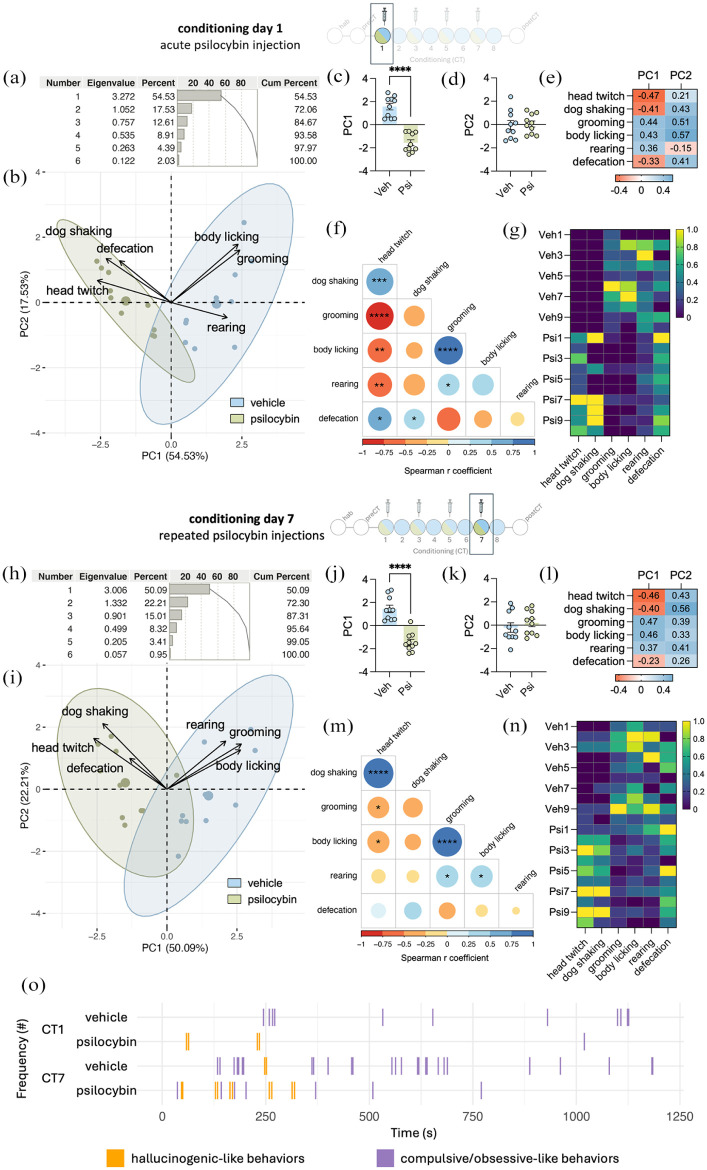
Alternate psilocybin administration induces stable and distinct behavioral clusters. Behavioral patterns for the acute effects of psilocybin (CT1): (a) eigenvalue and cumulative percentage for the PCA biplot, (b) PCA biplot, (c) PC1 (d) and PC2 values for the groups, (e) loading coefficient for PC1 and PC2, (f) correlation matrix, (g) heat map displaying the behavioral performance. Behavioral patterns for the repeated doses of psilocybin (CT7): (h) eigenvalue and cumulative percentage for the PCA biplot, (i) PCA biplot, (j) PC1 and (k) PC2 values for the groups, (l) loading coefficient for PC1 and PC2, (m) correlation matrix, and (n) heat map displaying the behavioral performance. (o) Time chart of the frequency of hallucinogenic- or compulsive/obsessive-like behaviors movements. *p < 0.05, **p < 0.01, ***p < 0.001, ****p < 0.0001.

To assess the relationship between behavioral outcomes and treatment, a PCA was applied. The PCA explained 72.06% of total results of the acute effects of psilocybin (CT1; Figure 3(a)). The PCA biplot analysis revealed that head twitch and wet-dog-shaking behaviors, along with pellets of defecation, were more closely associated with the psilocybin cluster. In contrast, body licking, grooming, and rearing behaviors were more correlated with the vehicle group (Figure 3(b)). An unpaired Student’s *t*-test demonstrated that PC1 was significantly more effective in separating the clusters (*t*_(18)_ = 8.123, *p* < 0.0001, Figure 3(c)) compared to PC2 (*t*_(18)_ = 0.190, *p* > 0.05, Figure 3(d)), showing that, for PC1, positive loading coefficients were more associated with the vehicle group, while negative loading coefficients were more associated with the psilocybin group Indeed, head twitch, wet-dog shaking, and defecation exhibited negative loading coefficient for PC1, whereas grooming, body licking, and rearing had positive loading coefficient (Figure 3(e)). A correlation matrix (Figure 3(f)) highlighted significant correlations between several behavioral measures, including head twitch × wet-dog shaking (*r* = 0.73, *p* < 0.001), head twitch × grooming (*r* = −0.77, *p* < 0.0001), head twitch × body licking (*r* = −0.58, *p* < 0.01), head twitch × rearing (*r* = −0.59, *p* < 0.01), head twitch × defecation (*r* = 0.55, *p* < 0.05), wet-dog shaking × rearing (*r* = −0.46, *p* < 0.05), grooming × body licking (*r* = 0.81, *p* < 0.001), grooming × rearing (*r* = 0.47, *p* < 0.05), grooming × defecation (*r* = −0.54, *p* < 0.05), and body licking × rearing (*r* = 0.46, *p* < 0.05). A heat map was built to further visualize the behavioral performance of each rat (Figure 3(g)).

PCA explained 72.30% of the total variance of CT7 (after receiving the 4th dose) results (Figure 3(h)). Like the acute effects, the PCA biplot analysis showed that head twitch, wet-dog shaking, and defecation were more strongly associated with the psilocybin cluster, while body licking, grooming, and rearing were more correlated with the vehicle group (Figure 3(i)). An unpaired Student’s *t*-test indicated that PC1 was significantly better at separating the clusters (*t*_(18)_ = 7.068, *p* < 0.0001, Figure 3(j)) compared to PC2 (*t*_(18)_ = 0.446, *p* > 0.05, Figure 3(k)), showing that, for PC1, positive loading coefficients were more associated with the vehicle group, while negative loading coefficients were more associated with the psilocybin group. Indeed, head twitch, wet-dog shaking, and defecation exhibited negative loading coefficient for PC1, whereas grooming, body licking, and rearing had positive loading coefficient (Figure 3(l)). The correlation matrix (Figure 3(m)) revealed statistical associations between behaviors, such as head twitch × wet-dog shaking (*r* = 0.89, *p* < 0.0001), head twitch × grooming (*r* = −0.46, *p* < 0.05), head twitch × body licking (*r* = −0.48, *p* < 0.05), grooming × body licking (*r* = 0.87, *p* < 0.0001), grooming × rearing (*r* = 0.16, *p* < 0.05), and body licking × rearing (*r* = 0.49, *p* < 0.05). A heat map displayed the behavioral performance of each rat (Figure 3(n)). The time chart (Figure 3(o)) highlights the frequency of hallucinogenic-like events (head twitch and wet-dog shaking), and compulsive or obsessive-like behaviors (grooming and body licking) performed on CT1 and CT7.

Taken these results together, behavioral clusters formed on the first conditioning day (CT1) remained consistent until the last psilocybin administration day (CT7), suggesting that alternate psilocybin administration induces stable and reproducible behavioral patterns over time, with no apparent development of tolerance to its behavioral effects.

### Behavioral changes induced by psilocybin are not long-lasting

**Figure 4.**
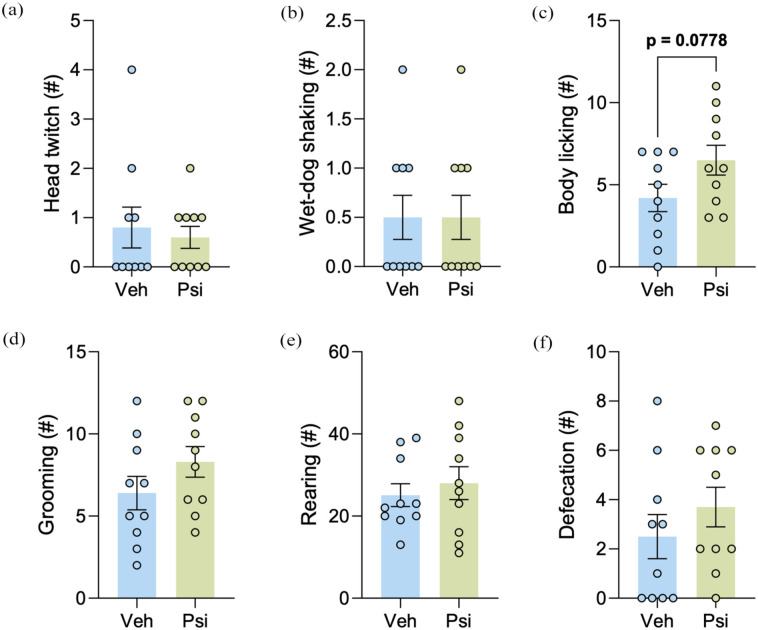
Behavioral assessment on the postCT day (48 hours after the last psilocybin injection, *n* = 10 each group). Analysis of (a) head twitch, (b) wet-dog shaking, (c) body licking, (d) grooming, (e) rearing, and (f) defecation pellets performed by each group. Unpaired Student’s *t*-test. Data are expressed as mean ± SEM.

To evaluate the long-lasting effect of psilocybin, behavioral assessment was conducted 2 days after the last psilocybin injection (postCT day). An unpaired Student’s *t*-test showed no differences between groups for head twitch (*t*_(18)_ = 0.4243; *p* > 0.05; Figure 4(a)), wet-dog shaking (*t*_(18)_ = 0.0000; *p* > 0.05; Figure 4(b)), body licking (*t*_(18)_ = 1.870; *p* > 0.05; Figure 4(c)), grooming (*t*_(18)_ = 1.380; *p* > 0.05; Figure 4(d)), rearing (*t*_(18)_ = 0.5957; *p* > 0.05; Figure 4(e)), and defecation pellets (*t*_(18)_ = 0.9962; *p* > 0.05; Figure 4(f)). These results reveal that the behavioral changes resulting from psilocybin administration are not long-lasting. Means and SEM are displayed in Table 3.

**Table 3.** Behavioral assessment on postCT day. Data are expressed as mean ± SEM.

Groups	Head twitch	Wet-dog shaking	Grooming	Body licking	Rearing	Defecation
Vehicle	0.80 ± 0.42	0.50 ± 0.22	6.40 ± 1.01	4.20 ± 0.83	25.1 ± 2.77	2.50 ± 0.89
Psilocybin	0.60 ± 0.22	0.50 ± 0.22	8.30 ± 0.93	6.50 ± 0.91	28.0 ± 4.00	3.70 ± 0.80

## Discussion

This study investigated the effects of high-dose psilocybin using the CPP paradigm. Our findings demonstrate that psilocybin at 10 mg/kg does not produce rewarding or aversive effects in male Sprague-Dawley rats. However, this administration regimen, compared to control animals, induced a distinctive behavioral pattern: increase in head-twitch and wet-dog-shaking responses, while reducing grooming and body-licking behaviors without changes in locomotor activity.

### Rewarding effects

It is well established that drugs of abuse, such as cocaine (Caffino et al., 2021; Camarini et al., 2019; Itzhak and Martin, 2002; Zhou et al., 2019), heroin (O’Neal et al., 2022), and ethanol (Carrara-Nascimento et al., 2014; Rae et al., 2018, 2024) induce CPP, demonstrating their rewarding effects, which are key contributors to the development and maintenance of substance use disorders. However, although psychedelics are not associated with the development of substance use disorder (Canal and Murnane, 2017; Nichols, 2016), LSD (Meehan and Schechter, 1998; Parker, 1996), ayahuasca (Cata-Preta et al., 2018; Reis et al., 2020), ketamine (Contó et al., 2022; Guo et al., 2016), and MDMA (Daza-Losada et al., 2007; García-Pardo et al., 2021; Roger-Sánchez et al., 2013) may produce rewarding effects and are classified as weakly reinforcing agents (NIDA, 2024). To assess the potential rewarding effects of psilocybin, we conducted an alternate-session CPP protocol, using a high-dose psilocybin during the conditioning sessions, and found that psilocybin did not induce CPP in male Sprague-Dawley rats. This finding aligns with Wang et al. (2023), who examined the effects of 1 mg/kg psilocin (the active metabolite of psilocybin) with a similar conditioning regimen, observing no rewarding effects. Notably, psilocybin is preferred to use in preclinical studies over psilocin because it offers greater stability and water solubility compared to psilocin, which is rapidly degrades when exposed to oxygen at room temperature (Wulff et al., 2023). Additionally, using the same conditioning regimen, ibogaine, a psychedelic extracted from the plant *Tabernanthe iboga Baill*, (10 or 30 mg/kg i.p.) also did not induce CPP (Henriques et al., 2021). However, using self-administration model, Fantegrossi et al. (2004) have reported that psychedelics like psilocybin, N,N-dimethyltryptamine, and mescaline can exhibit transient reinforcing effects in non-human primates with a history of MDMA self-administration.

Drugs of abuse primarily share a common pathway on reinforcing and addictive effects by increasing the firing of dopamine (DA) neurons in the ventral tegmental area leading to an increased DA release in the *nucleus accumbens* (Nutt et al., 2015; Volkow et al., 2011; Wise and Robble, 2020). However, while DA neurocircuitry plays a central role in addiction, it is also well-established that the serotonergic system contributes as a supportive mechanism (Canal and Murnane, 2017; Kirby et al., 2011; Müller and Homberg, 2015). Beyond their role as 5-HT_2A_R agonists, psychedelic substances also exhibit high affinity and functional activity at other serotonin receptors, including the 5-HT_2C_R (Canal and Murnane, 2017; Custodio et al., 2023; Erkizia-Santamaría et al., 2022: 2; Müller and Carey, 2006). Moreover, Canal et al. (2010) argue that contrasting effects have been reported between the 5-HT_2C_ and 5-HT_2A_ receptor subtypes. Specifically, 5-HT_2A_R activity is associated with enhanced DA neurotransmission, whereas 5-HT_2C_R activation inhibits DA release, thereby potentially reducing the risk of addiction.

### Hallucinogenic-like behaviors

Even though the present results indicated a lack of psilocybin-induced CPP, a more detailed analysis of the behavior during the conditioning sessions was made. We observed significant behavioral changes induced by psilocybin, allowing us to identify a distinct pattern through PCA analysis. Notably, the psilocybin treatment is linked to an increased head twitches and wet-dog-shaking responses, while reducing grooming, body licking and rearing behaviors.

Head twitches and wet-dog shaking are particularly relevant because they serve as primary behavioral markers to assess hallucinogenic-like effects in preclinical experiments (Canal and Morgan, 2012; de la Fuente Revenga et al., 2020; Halberstadt, 2020; Jaster and González-Maeso, 2023). In fact, 5-HT_2A_R agonists dose-dependently increase head twitch response (Jefferson et al., 2023; Wallach et al., 2023). Moreover, when pretreated with a 5-HT_2A_ antagonist ketanserin (Shao et al., 2021), MDL11939 (Erkizia-Santamaría et al., 2022), or genetically blocked as in 5-HT_2A_ knockout mice (Canal and Morgan, 2012; Halberstadt et al., 2011), the head twitch is abolished. However, this complex behavior is also mediated by 5-HT _1A_ and 5-HT_2C_ receptors and trace amine-associated receptors activity (Canal et al., 2010; Custodio et al., 2023; Shahar et al., 2022; Zhu et al., 2024). In addition, Buchborn et al. (2023) and Brockett and Francis (2024) showed that shaking behaviors, including wet-dog shaking, are likely mediated by serotonin 2A receptors on cortical pyramidal cells.

These findings align with previous studies demonstrating that wet-dog-shaking behavior is closely tied to serotonergic activity (Bedard and Pycock, 1977; Yap and Taylor, 1983). Interestingly, the expression of wet-dog shaking appears to involve specific brain regions, including the brainstem and diencephalon, further emphasizing its role as a behavioral model for central serotonergic activity (Bedard and Pycock, 1977).

Every other day injection of psilocybin progressively increased the frequency of head twitches and wet-dog-shaking responses. Interestingly, Smith et al. (2014) investigated the effects of different injection regimens of 1 mg/kg i.p. DOI (2,5-dimethoxy-4-iodoamphetamine) in mice. They found that daily or an alternate-day administration led to tolerance and a reduction in head twitches over time, whereas no changes were observed with weekly administration. However, this tolerance might be reversed by increasing the dose on the second consecutive day of injection (de la Fuente Revenga et al., 2022). At a high dose (5 mg/kg i.p.), DOI injection exhibited tolerance when administered daily, but no changes were observed with administration every 48 hours. Similarly, tolerance was observed with daily administration of 0.2 mg/kg i.p. LSD for four consecutive days. In contrast, when the tryptamines N,N-dipropyltryptamine (3 mg/kg i.p.) or N,N-diisopropyltryptamine (10 mg/kg i.p.) was administered for four consecutive days, no tolerance or sensitization was observed (Smith et al., 2014). Therefore, the pattern of behavioral effects and changes in subsequent sensitivity depend on the dose, frequency of administration, and the time elapsed since the last injection. To our knowledge, no studies have examined the effects of psilocybin administered every other day at high doses to determine whether sensitivity is maintained over time.

### Anti-compulsive effects

We found that psilocybin decreases the frequency of grooming and body licking behaviors. Self-grooming is a complex behavior that serves critical biological functions and is particularly relevant to various pathological conditions (Xie et al., 2022). Generally, grooming behavior is followed by a chain pattern, which is followed by body licking (Smolinsky et al., 2009). While our results align with the effects of MDMA (Pálenícek et al., 2007), acute LSD injection has been found to increase grooming frequency or duration (Kyzar et al., 2016; Rodriguiz et al., 2021). Brownstien et al. (2024) and Gattuso et al. (2024) used a SAPAP3 knockout mouse model of compulsive-like behavior and also showed that psilocybin reduced grooming frequency. Interestingly, substances associated with abuse, such as amphetamines and cocaine, have been shown to decrease the frequency or duration of grooming (Carey et al., 2005; Earley and Leonard, 1978; Moy et al., 2014).

Moreover, grooming behavior has been shown to respond to various genetic and pharmacological interventions and is highly sensitive to stress (Audet et al., 2006; Jia et al., 2023; Mu et al., 2020; Smolinsky et al., 2009) and anxiety (Kalueff and Tuohimaa, 2005; Nin et al., 2012). However, the impact of psilocybin in anxiety in a preclinical model seems very controversial. Some studies suggest that psilocybin may induce anxiogenic-like behavior (Harari et al., 2024), while others report anxiolytic response (Hibicke et al., 2020; Jones et al., 2023).

Recent preclinical studies have further highlighted psilocybin’s therapeutic potential in addressing substance use disorders. In self-administration models, psilocybin has been shown to reduce ethanol self-administration (Jeanblanc et al., 2024), as well as reduce heroin-seeking behavior (Floris et al., 2024). Additionally, psilocybin suppressed the acquisition of methamphetamine-induced CPP through D_2_R-mediated ERK signaling (Wang et al., 2023).

Therapeutic outcomes observed with psilocybin treatment align with effects reported for other psychedelic substances. In the CPP paradigm, the rewarding effects of ethanol were attenuated or blocked by ibogaine (Henriques et al., 2021) and ayahuasca (Cata-Preta et al., 2018; Gianfratti et al., 2022). A 1:1 ratio of cannabidiol:tetrahydrocannabinol attenuated methamphetamine-CPP (Nukitram et al., 2023). Ayahuasca prevented the methylphenidate-CPP (Reis et al., 2020).

In conclusion, our study demonstrates that conditioning protocol with 4 doses of psilocybin administered on alternate days does not induce rewarding effects in rats but generates a unique behavioral profile, characterized by an increase in head-twitch and wet-dog-shaking responses and a decrease in rearing, grooming, and body-licking behaviors, with no effect on locomotor activity. However, post-conditioning assessment revealed no long-lasting behavioral effects, supporting current perspectives on the tolerability and safety profile of psilocybin. Further research exploring alternative models of addiction-like behavior, particularly those involving chronic exposure, and incorporating molecular and neurocircuitry analyses, is essential to characterize in depth the rewarding-related effects associated with psilocybin. However, our findings bring valuable insights to the understanding of behavioral effects of psychedelics, offering a foundation for future studies on their therapeutic potential and safety in addressing substance use disorders.

## References

[bibr1-02698811251368361] Audet MC Goulet S Doré FY (2006) Repeated subchronic exposure to phencyclidine elicits excessive atypical grooming in rats. Behavioural Brain Research 167(1): 103–110. 16257455 10.1016/j.bbr.2005.08.026

[bibr2-02698811251368361] Bedard P Pycock CJ (1977) ‘Wet-Dog’ shake behaviour in the rat: A possible quantitative model of central 5-hydroxytryptamine activity. Neuropharmacology 16(10): 663–670. 304190 10.1016/0028-3908(77)90117-4

[bibr3-02698811251368361] Brockett AT Francis NA (2024) Psilocybin biphasically modulates cortical and behavioral activity in mice. bioRxiv. DOI: 10.1101/2024.01.18.576229.

[bibr4-02698811251368361] Brownstien M Lazar M Botvinnik A , et al. (2024) Striking long term beneficial effects of single dose psilocybin and psychedelic mushroom extract in the SAPAP3 rodent model of OCD-like excessive self-grooming. bioRxiv. DOI: 10.1101/2024.06.25.600634. PMC11835722 39394457

[bibr5-02698811251368361] Bruno V Wiazowski Spelta LE Durão AC , et al. (2022) Psychedelics and mental health: An alternative strategy to treat mental impairments triggered or aggravated by COVID-19. Alternative Therapies in Health and Medicine 28(4): 40–43. 35427236

[bibr6-02698811251368361] Buchborn T Lyons T Song C , et al. (2023) Cortical correlates of psychedelic-induced shaking behavior revealed by voltage imaging. International Journal of Molecular Sciences 24(11): 9463. 37298417 10.3390/ijms24119463 PMC10253917

[bibr7-02698811251368361] Caffino L Moro F Mottarlini F , et al. (2021). Repeated exposure to cocaine during adolescence enhances the rewarding threshold for cocaine-conditioned place preference in adulthood. Addiction Biology 26(5): e13012. 10.1111/adb.13012 33511707

[bibr8-02698811251368361] Camarini R Hoffmann LB Suarez A , et al. (2019) Cocaine-induced behavioral sensitization is greater in adolescent than in adult mice and heightens cocaine-induced conditioned place preference in adolescents. Pharmacology, Biochemistry, and Behavior 181: 60–68. 31004629 10.1016/j.pbb.2019.04.005

[bibr9-02698811251368361] Canal CE Morgan D (2012) Head-twitch response in rodents induced by the hallucinogen 2,5-dimethoxy-4-iodoamphetamine: A comprehensive history, a re-evaluation of mechanisms, and its utility as a model. Drug Testing and Analysis 4(7–8): 556–576. 22517680 10.1002/dta.1333 PMC3722587

[bibr10-02698811251368361] Canal CE Murnane KS (2017) The serotonin 5-HT2C receptor and the non-addictive nature of classic hallucinogens. Journal of Psychopharmacology 31(1): 127–143. 27903793 10.1177/0269881116677104 PMC5445387

[bibr11-02698811251368361] Canal CE Olaghere da Silva UB Gresch PJ , et al. (2010) The serotonin 2C receptor potently modulates the head-twitch response in mice induced by a phenethylamine hallucinogen. Psychopharmacology 209(2): 163–174. 20165943 10.1007/s00213-010-1784-0 PMC2868321

[bibr12-02698811251368361] Carey RJ DePalma G Damianopoulos E (2005) Evidence for Pavlovian conditioning of cocaine-induced responses linked to emotional behavioral effects. Pharmacology Biochemistry and Behavior 80(1): 123–134. 15652388 10.1016/j.pbb.2004.10.012

[bibr13-02698811251368361] Carhart-Harris R Giribaldi B Watts R , et al. (2021) Trial of psilocybin versus escitalopram for depression. The New England Journal of Medicine 384(15): 15. 10.1056/NEJMoa2032994 33852780

[bibr14-02698811251368361] Carrara-Nascimento PF Olive MF Camarini R (2014) Ethanol pre-exposure during adolescence or adulthood increases ethanol intake but ethanol-induced conditioned place preference is enhanced only when pre-exposure occurs in adolescence. Developmental Psychobiology 56(1): 36–48. 23129501 10.1002/dev.21089

[bibr15-02698811251368361] Cata-Preta EG Serra YA Moreira-Junior EDC , et al. (2018) Ayahuasca and its DMT- and β-carbolines – Containing ingredients block the expression of ethanol-induced conditioned place preference in mice: Role of the treatment environment. Frontiers in Pharmacology 9: 561. 29896106 10.3389/fphar.2018.00561 PMC5986901

[bibr16-02698811251368361] Chen Y Liu J Yao Y , et al. (2023) Rearing behaviour in the mouse behavioural pattern monitor distinguishes the effects of psychedelics from those of lisuride and TBG. Frontiers in Pharmacology 14: 1021729. 36874002 10.3389/fphar.2023.1021729 PMC9978355

[bibr17-02698811251368361] Contó MB Pautassi RM Camarini R (2022) Rewarding and antidepressant properties of ketamine and ethanol: effects on the brain-derived neurotrophic factor and TrkB and p75NTR receptors. Neuroscience 493: 1–14. 35469972 10.1016/j.neuroscience.2022.04.015

[bibr18-02698811251368361] Custodio RJP Ortiz DM Lee HJ , et al. (2023) Serotonin 2C receptors are also important in head-twitch responses in male mice. Psychopharmacology 242(7): 1585–1605. 37882810 10.1007/s00213-023-06482-9

[bibr19-02698811251368361] Daza-Losada M Ribeiro Do Couto B Manzanedo C , et al. (2007) Rewarding effects and reinstatement of MDMA-induced CPP in adolescent mice. Neuropsychopharmacology 32(8): 1750–1759. 17299518 10.1038/sj.npp.1301309

[bibr20-02698811251368361] de la Fuente Revenga M Jaster AM McGinn J , et al. (2022) Tolerance and cross-tolerance among psychedelic and nonpsychedelic 5-HT2A receptor agonists in mice. ACS Chemical Neuroscience 13(16): 2436–2448. 35900876 10.1021/acschemneuro.2c00170 PMC10411500

[bibr21-02698811251368361] de la Fuente Revenga M Vohra HZ González-Maeso J (2020) Automated quantification of head-twitch response in mice via ear tag reporter coupled with biphasic detection. Journal of Neuroscience Methods 334: 108595. 31954738 10.1016/j.jneumeth.2020.108595 PMC7363508

[bibr22-02698811251368361] Dinis-Oliveira RJ (2017) Metabolism of psilocybin and psilocin: Clinical and forensic toxicological relevance. Drug Metabolism Reviews 49(1): 84–91. 28074670 10.1080/03602532.2016.1278228

[bibr23-02698811251368361] Earley CJ Leonard BE (1978) Behavioural studies on the effects of d-amphetamine and estradiol benzoate alone and in combination. Psychopharmacology 56(2): 179–183. 417368 10.1007/BF00431846

[bibr24-02698811251368361] Erkizia-Santamaría I Alles-Pascual R Horrillo I , et al. (2022) Serotonin 5-HT2A, 5-HT2c and 5-HT1A receptor involvement in the acute effects of psilocybin in mice. In vitro pharmacological profile and modulation of thermoregulation and head-twich response. Biomedicine & Pharmacotherapy 154: 113612. 36049313 10.1016/j.biopha.2022.113612

[bibr25-02698811251368361] Fantegrossi WE Woods JH Winger G (2004) Transient reinforcing effects of phenylisopropylamine and indolealkylamine hallucinogens in rhesus monkeys. Behavioural Pharmacology 15(2): 149. 15096915 10.1097/00008877-200403000-00007

[bibr26-02698811251368361] Floris G Dabrowski KR Zanda MT , et al. (2024) Psilocybin reduces heroin seeking behavior and modulates inflammatory gene expression in the nucleus accumbens and prefrontal cortex of male rats. Molecular Psychiatry 30(5): 1801–1816. 39433903 10.1038/s41380-024-02788-y PMC12015112

[bibr27-02698811251368361] García-Pardo MP De la Rubia Ortí JE Calpe-López C , et al. (2021) Role of acute social stress in the rewarding effects of MDMA in adolescent mice. Behavioural Brain Research 410: 113348. 33971245 10.1016/j.bbr.2021.113348

[bibr28-02698811251368361] Gattuso JJ Wilson C Hannan AJ , et al. (2024) Psilocybin reduces grooming in the SAPAP3 knockout mouse model of compulsive behaviour. bioRxiv. DOI: 10.1101/2024.10.23.619763. 39489287

[bibr29-02698811251368361] Gevins A Smith ME McEvoy LK (2002) Tracking the cognitive pharmacodynamics of psychoactive substances with combinations of behavioral and neurophysiological measures. Neuropsychopharmacology 26(1): 27–39. 11751030 10.1016/S0893-133X(01)00300-1

[bibr30-02698811251368361] Gianfratti B Tabach R Sakalem ME , et al. (2022) Ayahuasca blocks ethanol preference in an animal model of dependence and shows no acute toxicity. Journal of Ethnopharmacology 285: 114865. 34822961 10.1016/j.jep.2021.114865

[bibr31-02698811251368361] Goodwin GM Aaronson ST Alvarez O , et al. (2022) Single-dose psilocybin for a treatment-resistant episode of major depression. The New England Journal of Medicine 387(18): 1637–1648. 36322843 10.1056/NEJMoa2206443

[bibr32-02698811251368361] Greenacre M Groenen PJF Hastie T , et al. (2022) Principal component analysis. Nature Reviews Methods Primers 2(1): 1–21.

[bibr33-02698811251368361] Griffiths RR Johnson MW Richards WA , et al. (2011) Psilocybin occasioned mystical-type experiences: Immediate and persisting dose-related effects. Psychopharmacology 218(4): 649–665. 21674151 10.1007/s00213-011-2358-5 PMC3308357

[bibr34-02698811251368361] Guo R Tang Q Ye Y , et al. (2016) Effects of gender on ketamine-induced conditioned placed preference and urine metabonomics. Regulatory Toxicology and Pharmacology: RTP 77: 263–274. 26995028 10.1016/j.yrtph.2016.03.007

[bibr35-02698811251368361] Halberstadt AL (2020) Automated detection of the head-twitch response using wavelet scalograms and a deep convolutional neural network. Scientific Reports 10(1): 8344. 32433580 10.1038/s41598-020-65264-x PMC7239849

[bibr36-02698811251368361] Halberstadt AL Koedood L Powell SB , et al. (2011) Differential contributions of serotonin receptors to the behavioral effects of indoleamine hallucinogens in mice. Journal of Psychopharmacology 25(11): 1548–1561. 21148021 10.1177/0269881110388326 PMC3531560

[bibr37-02698811251368361] Harari R Chatterjee I Getselter D , et al. (2024) Psilocybin induces acute anxiety and changes in amygdalar phosphopeptides independently from the 5-HT2A receptor. iScience 27(5): 109686. 38660396 10.1016/j.isci.2024.109686 PMC11039401

[bibr38-02698811251368361] Henriques GM Anjos-Santos A Rodrigues IRS , et al. (2021) Ibogaine blocks cue- and drug-induced reinstatement of conditioned place preference to ethanol in male mice. Frontiers in Pharmacology 12: 739012. 34621171 10.3389/fphar.2021.739012 PMC8490685

[bibr39-02698811251368361] Hibicke M Landry AN Kramer HM , et al. (2020) Psychedelics, but not ketamine, produce persistent antidepressant-like effects in a rodent experimental system for the study of depression. ACS Chemical Neuroscience 11(6): 864–871. 32133835 10.1021/acschemneuro.9b00493

[bibr40-02698811251368361] Higgins GA Carroll NK Brown M , et al. (2021) Low doses of psilocybin and ketamine enhance motivation and attention in poor performing rats: Evidence for an antidepressant property. Frontiers in Pharmacology 12: 640241. 33716753 10.3389/fphar.2021.640241 PMC7952974

[bibr41-02698811251368361] Itzhak Y Martin JL (2002) Cocaine-induced conditioned place preference in mice: Induction, extinction and reinstatement by related psychostimulants. Neuropsychopharmacology 26(1): 130–134. 11751040 10.1016/S0893-133X(01)00303-7

[bibr42-02698811251368361] Jaster AM González-Maeso J (2023) Automated detection of psychedelic-induced head-twitch response in mice. Methods in Molecular Biology (Clifton, N.J.) 2687: 65–76. 10.1007/978-1-0716-3307-6_6 37464163

[bibr43-02698811251368361] Jeanblanc J Bordy R Fouquet G , et al. (2024) Psilocybin reduces alcohol self-administration via selective left nucleus accumbens activation in rats. Brain 147(11): 3780–3788. 38703387 10.1093/brain/awae136

[bibr44-02698811251368361] Jefferson SJ Gregg I Dibbs M , et al. (2023) 5-MeO-DMT modifies innate behaviors and promotes structural neural plasticity in mice. Neuropsychopharmacology 48(9): 1257–1266. 37015972 10.1038/s41386-023-01572-w PMC10354037

[bibr45-02698811251368361] Jia T Chen J Wang YD , et al. (2023) A subthalamo-parabrachial glutamatergic pathway is involved in stress-induced self-grooming in mice. Acta Pharmacologica Sinica 44(11): 2169–2183. 37322164 10.1038/s41401-023-01114-6 PMC10618182

[bibr46-02698811251368361] Jones NT Zahid Z Grady SM , et al. (2023) Transient elevation of plasma glucocorticoids supports psilocybin-induced anxiolysis in mice. ACS Pharmacology & Translational Science 6(8): 1221–1231. 37588757 10.1021/acsptsci.3c00123 PMC10425994

[bibr47-02698811251368361] Kalueff AV Tuohimaa P (2005) Mouse grooming microstructure is a reliable anxiety marker bidirectionally sensitive to GABAergic drugs. European Journal of Pharmacology 508(1-3): 147–153. 15680265 10.1016/j.ejphar.2004.11.054

[bibr48-02698811251368361] Kirby LG Zeeb FD Winstanley CA (2011) Contributions of serotonin in addiction vulnerability. Neuropharmacology 61(3): 421–432. 21466815 10.1016/j.neuropharm.2011.03.022 PMC3110503

[bibr49-02698811251368361] Kyzar EJ Stewart AM Kalueff AV (2016) Effects of LSD on grooming behavior in serotonin transporter heterozygous (Sert+/–) mice. Behavioural Brain Research 296: 47–52. 26340513 10.1016/j.bbr.2015.08.018

[bibr50-02698811251368361] Lowe H Toyang N Steele B , et al. (2021) The therapeutic potential of psilocybin. Molecules 26(10): 2948. 34063505 10.3390/molecules26102948 PMC8156539

[bibr51-02698811251368361] Madsen MK Fisher PM Burmester D , et al. (2019). Psychedelic effects of psilocybin correlate with serotonin 2A receptor occupancy and plasma psilocin levels. Neuropsychopharmacology 44(7); 1328–1334. 30685771 10.1038/s41386-019-0324-9 PMC6785028

[bibr52-02698811251368361] McClure-Begley TD Roth BL (2022) The promises and perils of psychedelic pharmacology for psychiatry. Nature Reviews. Drug Discovery 21(6): 463–473. 35301459 10.1038/s41573-022-00421-7

[bibr53-02698811251368361] McCulloch DE-W Grzywacz MZ Madsen MK , et al. (2022) Psilocybin-induced mystical-type experiences are related to persisting positive effects: A quantitative and qualitative report. Frontiers in Pharmacology 13: 841648. 35355714 10.3389/fphar.2022.841648 PMC8959755

[bibr54-02698811251368361] Meehan SM Schechter MD (1998) LSD produces conditioned place preference in male but not female fawn hooded rats. Pharmacology Biochemistry and Behavior 59(1): 105–108. 9443543 10.1016/s0091-3057(97)00391-2

[bibr55-02698811251368361] Moy SS Riddick NV Nikolova VD , et al. (2014) Repetitive behavior profile and supersensitivity to amphetamine in the C58/J mouse model of autism. Behavioural Brain Research 259: 200–214. 24211371 10.1016/j.bbr.2013.10.052 PMC3883138

[bibr56-02698811251368361] Mu MD Geng HY Rong KL , et al. (2020) A limbic circuitry involved in emotional stress-induced grooming. Nature Communications 11(1): 2261. 10.1038/s41467-020-16203-x PMC7210270 32385304

[bibr57-02698811251368361] Müller CP Carey RJ (2006) Intracellular 5-HT 2C-receptor dephosphorylation: A new target for treating drug addiction. Trends in Pharmacological Sciences 27(9): 455–458. 16876260 10.1016/j.tips.2006.07.003

[bibr58-02698811251368361] Müller CP Homberg JR (2015) The role of serotonin in drug use and addiction. Behavioural Brain Research 277: 146–192. 24769172 10.1016/j.bbr.2014.04.007

[bibr59-02698811251368361] Murnane KS (2018) The renaissance in psychedelic research: What do preclinical models have to offer. Progress in Brain Research 242: 25–67. 30471682 10.1016/bs.pbr.2018.08.003

[bibr60-02698811251368361] Nair AB Jacob S (2016) A simple practice guide for dose conversion between animals and human. Journal of Basic and Clinical Pharmacy 7(2): 27. 27057123 10.4103/0976-0105.177703 PMC4804402

[bibr61-02698811251368361] Nichols DE (2016) Psychedelics. Pharmacological Reviews 68(2): 264–355. 26841800 10.1124/pr.115.011478 PMC4813425

[bibr62-02698811251368361] NIDA (2024) Psychedelic and dissociative drugs. Available at: https://nida.nih.gov/research-topics/psychedelic-dissociative-drugs (accessed September 2025).

[bibr63-02698811251368361] Nin MS Couto-Pereira NS Souza MF , et al. (2012) Anxiolytic effect of clonazepam in female rats: Grooming microstructure and elevated plus maze tests. European Journal of Pharmacology 684(1-3): 95–101. 22487059 10.1016/j.ejphar.2012.03.038

[bibr64-02698811251368361] Nukitram J Kumarnsit E Cheaha D (2023) A 1:1 ratio of cannabidiol: Tetrahydrocannabinol attenuates methamphetamine conditioned place preference in mice: A prospective study of antidopaminergic mechanism. Brain Research Bulletin 192: 47–55. 36336144 10.1016/j.brainresbull.2022.11.003

[bibr65-02698811251368361] Nutt D (2019) Psychedelic drugs-a new era in psychiatry? Dialogues in Clinical Neuroscience 21(2): 139–147. 31636488 10.31887/DCNS.2019.21.2/dnutt PMC6787540

[bibr66-02698811251368361] Nutt DJ Lingford-Hughes A Erritzoe D , et al. (2015). The dopamine theory of addiction: 40 years of highs and lows. Nature Reviews. Neuroscience 16(5): 305–312. 25873042 10.1038/nrn3939

[bibr67-02698811251368361] O’Neal TJ Bernstein MX MacDougall DJ , et al. (2022) A conditioned place preference for heroin is signaled by increased dopamine and direct pathway activity and decreased indirect pathway activity in the nucleus accumbens. Journal of Neuroscience 42(10): 2011–2024. 35031576 10.1523/JNEUROSCI.1451-21.2021 PMC8916759

[bibr68-02698811251368361] Pálenícek T Hlinák Z Bubeníková-Valesová V , et al. (2007) An analysis of spontaneous behavior following acute MDMA treatment in male and female rats. Neuro Endocrinology Letters 28(6): 781–788. 18063949

[bibr69-02698811251368361] Parker LA (1996) LSD produces place preference and flavor avoidance but does not produce flavor aversion in rats. Behavioral Neuroscience 110(3): 503–508. 8888996 10.1037//0735-7044.110.3.503

[bibr70-02698811251368361] Rae M Gomes I Spelta LEW , et al. (2024) Environmental enrichment enhances ethanol preference over social reward in male swiss mice: Involvement of oxytocin-dopamine interactions. Neuropharmacology 253: 109971. 38705568 10.1016/j.neuropharm.2024.109971 PMC11145911

[bibr71-02698811251368361] Rae M Zanos P Georgiou P , et al. (2018) Environmental enrichment enhances conditioned place preference to ethanol via an oxytocinergic-dependent mechanism in male mice. Neuropharmacology 138: 267–274. 29908241 10.1016/j.neuropharm.2018.06.013

[bibr72-02698811251368361] Reis HS Rodrigues IRS Anjos-Santos A , et al. (2020) Ayahuasca blocks the reinstatement of methylphenidate-induced conditioned place preference in mice: Behavioral and brain Fos expression evaluations. Psychopharmacology 237(11): 3269–3281. 32676773 10.1007/s00213-020-05609-6

[bibr73-02698811251368361] Rodriguiz RM Nadkarni V Means CR , et al. (2021) LSD-stimulated behaviors in mice require β-arrestin 2 but not β-arrestin 1. Scientific Reports 11(1): 17690. 34480046 10.1038/s41598-021-96736-3 PMC8417039

[bibr74-02698811251368361] Roger-Sánchez C Aguilar MA Manzanedo C , et al. (2013) Neurochemical substrates of MDMA reward: Effects of the inhibition of serotonin reuptake on the acquisition and reinstatement of MDMA-induced CPP. Current Pharmaceutical Design 19(40): 7050–7064. 23574442 10.2174/138161281940131209143632

[bibr75-02698811251368361] Shahar O Botvinnik A Esh-Zuntz N , et al. (2022) Role of 5-HT2A, 5-HT2C, 5-HT1A and TAAR1 receptors in the head twitch response induced by 5-hydroxytryptophan and psilocybin: Translational implications. International Journal of Molecular Sciences 23(22): 22. 10.3390/ijms232214148 PMC9698447 36430623

[bibr76-02698811251368361] Shao L-X Liao C Gregg I , et al. (2021) Psilocybin induces rapid and persistent growth of dendritic spines in frontal cortex in vivo. Neuron 109(16): 2535–2544.e4. 10.1016/j.neuron.2021.06.008 PMC8376772 34228959

[bibr77-02698811251368361] Smith DA Bailey JM Williams D , et al. (2014) Tolerance and cross-tolerance to head twitch behavior elicited by phenethylamine- and tryptamine-derived hallucinogens in mice. Journal of Pharmacology and Experimental Therapeutics 351(3): 485–491. 25271256 10.1124/jpet.114.219337 PMC4309922

[bibr78-02698811251368361] Smolinsky AN Bergner CL LaPorte JL , et al. (2009) Analysis of grooming behavior and its utility in studying animal stress, anxiety, and depression. In: Gould TD (ed.) Mood and Anxiety Related Phenotypes in Mice: Characterization Using Behavioral Tests. Totowa, NJ: Humana Press, pp. 21–36.

[bibr79-02698811251368361] Tan PZ Baldwin RM Van Dyck CH , et al. (1999) Characterization of radioactive metabolites of 5-HT2A receptor PET ligand [18F]altanserin in human and rodent. Nuclear Medicine and Biology 26(6): 601–608. 10587097 10.1016/s0969-8051(99)00022-0

[bibr80-02698811251368361] Tzschentke TM (1998) Measuring reward with the conditioned place preference paradigm: A comprehensive review of drug effects, recent progress and new issues. Progress in Neurobiology 56(6): 613–672. 9871940 10.1016/s0301-0082(98)00060-4

[bibr81-02698811251368361] Volkow ND Wang G-J Fowler JS , et al. (2011) Addiction: Beyond dopamine reward circuitry. Proceedings of the National Academy of Sciences of the United States of America 108(37): 15037–15042. 21402948 10.1073/pnas.1010654108 PMC3174598

[bibr82-02698811251368361] Wallach J Cao AB Calkins MM , et al. (2023) Identification of 5-HT2A receptor signaling pathways associated with psychedelic potential. Nature Communications 14(1): 8221. 10.1038/s41467-023-44016-1 PMC10724237 38102107

[bibr83-02698811251368361] Wang J Liang M Shang Q , et al. (2023) Psilocin suppresses methamphetamine-induced hyperlocomotion and acquisition of conditioned place preference via D2R-mediated ERK signaling. CNS Neuroscience & Therapeutics 29(3): 831–841. 36627756 10.1111/cns.14054 PMC9928547

[bibr84-02698811251368361] Wise RA Robble MA (2020) Dopamine and addiction. Annual Review of Psychology 71: 79–106. 10.1146/annurev-psych-010418-103337 31905114

[bibr85-02698811251368361] Wulff AB Nichols CD Thompson SM (2023) Preclinical perspectives on the mechanisms underlying the therapeutic actions of psilocybin in psychiatric disorders. Neuropharmacology 231: 109504. 36921889 10.1016/j.neuropharm.2023.109504

[bibr86-02698811251368361] Xie Z Li D Cheng X , et al. (2022) A brain-to-spinal sensorimotor loop for repetitive self-grooming. Neuron 110(5): 874–890.e7. 10.1016/j.neuron.2021.11.028 34932943

[bibr87-02698811251368361] Yap CY Taylor DA (1983) Involvement of 5-HT2 receptors in the wet-dog shake behaviour induced by 5-hydroxytryptophan in the rat. Neuropharmacology 22(7): 801–804. 6604883 10.1016/0028-3908(83)90123-5

[bibr88-02698811251368361] Yates JR (2023) Quantifying conditioned place preference: A review of current analyses and a proposal for a novel approach. Frontiers in Behavioral Neuroscience 17: 1256764. 37693282 10.3389/fnbeh.2023.1256764 PMC10484009

[bibr89-02698811251368361] Zhou Y Zhu H Liu Z , et al. (2019) A ventral CA1 to nucleus accumbens core engram circuit mediates conditioned place preference for cocaine. Nature Neuroscience 22(12): 1986–1999. 31719672 10.1038/s41593-019-0524-y

[bibr90-02698811251368361] Zhu H Wang L Wang X , et al. (2024) 5-hydroxytryptamine 2C/1A receptors modulate the biphasic dose response of the head twitch response and locomotor activity induced by DOM in mice. Psychopharmacology 241(11): 2315–2330. 38916640 10.1007/s00213-024-06635-4

